# Vulvar Adenocarcinoma With Pagetoid Spread: A Case Report

**DOI:** 10.1002/cnr2.70241

**Published:** 2025-06-04

**Authors:** Kazuaki Nishimura, Seiji Kagami, Haruka Kajio, Tamaki Wada, Naoyuki Toki, Kiyoshi Yoshino

**Affiliations:** ^1^ Department of Obstetrics and Gynecology School of Medicine, University of Occupational and Environmental Health Kitakyushu Japan; ^2^ Department of Obstetrics and Gynecology Kyushu Rosai Hospital Kitakyushu Japan

**Keywords:** CDX2, CK20, pagetoid spread, Paget's disease

## Abstract

**Background:**

In rare cases, cancer cells adjacent to the skin migrate intraepithelially and reach the epidermis, presenting a histological appearance similar to that of Paget's disease; this phenomenon is termed pagetoid spread. Herein, we report a case of vulvar adenocarcinoma with pagetoid spread, in which the primary lesion could not be identified outside the vulva.

**Case:**

An 84‐year‐old woman, presented with a 5‐cm tumor in the right vulva. Histopathological examination revealed atypical Paget cells. Immunohistochemical examination revealed that the cells were positive for CK20 and CK7 and negative for CDX2 and GCDFP15. Thus, it was determined that the tumor was not a primary extramammary Paget's disease, but a secondary extramammary Paget's disease with pagetoid spread. There were no masses in the anus, rectum, urinary tract, or vagina. Computed tomography scan revealed a right vulvar tumor and lymph node enlargement around the abdominal aorta, and the tumor was diagnosed as lymph node metastasis of vulvar adenocarcinoma associated with extramammary Paget's disease.

**Conclusion:**

We report a rare case of vulvar adenocarcinoma with pagetoid spread.

## 1 Introduction

Extramammary Paget's disease (EMPD) is characterized by the proliferation and progression of atypical Paget's cells within the epidermis. It is broadly divided into primary and secondary EMPD, according to the primary lesion [1]. Primary EMPD is thought to originate from apocrine sweat glands, as it occurs most frequently in the vulva, axilla, and perianal area. Secondary EMPD is characterized by the migration of cancer cells from an organ adjacent to the skin through the epithelium to reach the epidermis and presents as intraepidermal cancer, a phenomenon called “pagetoid spread.” It usually refers to the progression of anal canal, rectal, bladder, or gynecological cancer to the vulvar skin. The clinical and pathological features of primary EMPD and secondary EMPD with pagetoid spread are similar, but the treatment approaches are completely different. Therefore, their differentiation using immunohistochemical staining is important [2]. Secondary EMPD of the vulva, also known as pagetoid spread, diagnosed using immunohistochemical staining, essentially develops from a site other than the vulva [3, 4, 5], and there are no reports of vulvar adenocarcinoma with pagetoid spread in which no mass was identified apart from vulvar cancer. Herein, we report a case of vulvar adenocarcinoma with pagetoid spread, in which the primary lesion could not be identified outside the vulva.

## 2 Case

**FIGURE 1:**
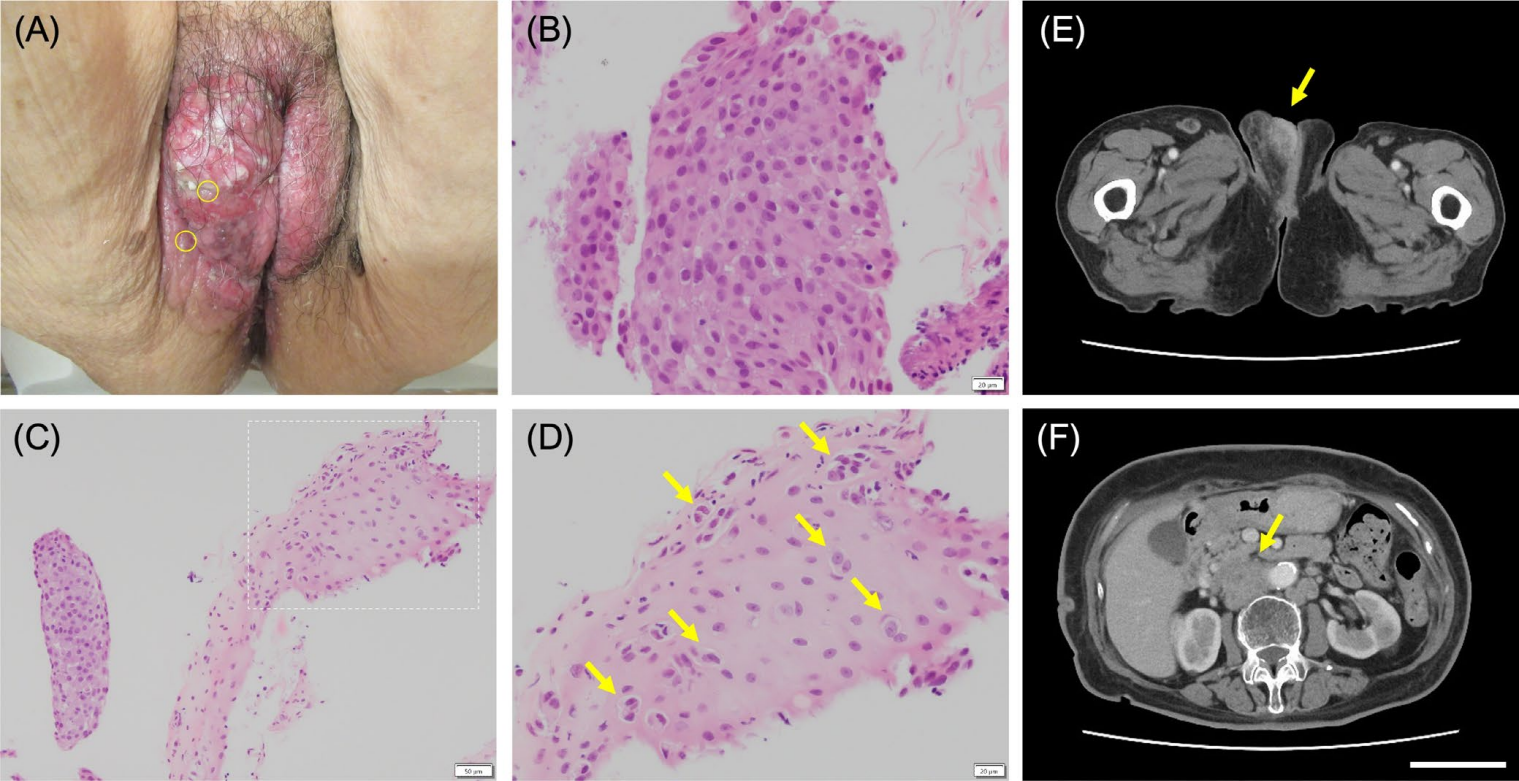
Symptoms, pathology, and abdominal computed tomography (CT) scans of vulvar cancer (A) Clinical examination showing a tumor with an irregular surface was found on the right vulva, measuring 5 cm in maximal dimension. (B) The tissue biopsied from the center of the tumor contained atypical Paget cells. Hematoxylin and eosin stain; 10× magnification. (C) Similarly, progression of atypical cells was observed in the tissue from the edge. Hematoxylin and eosin stain; 20× maginification. (D) Enlargement of the area enclosed by the dotted line in (C). The tissue showed the progression of atypical Paget cells in a small alveolar or scattered pattern within the non‐neoplastic squamous epithelium(arrow). Hematoxylin and eosin stain; 10× maginification. (E) CT reveals a well‐enhancing lesion (arrow) at the right vulvar portion. Enlarged bilateral inguinal lymph nodes are found. (F) Multiple enlarged abdominal para‐aortic lymph nodes were observed(arrow).

An 84‐year‐old woman visited Kyushu Rosai Hospital with a vulvar mass. Her medical history included Alzheimer's dementia, hypertension, and diabetes. A 5 × 4 cm raised mass with an irregular surface was found on the right vulva (Figure 1A). There were no obvious vaginal lesions. No obvious masses were observed in the urethra. There was no tumor around the anus, but a white lesion was found on the vulva, 1 cm lateral to the anal line. No mass was palpable during rectal examination. Enlargement of the right inguinal lymph node was palpable. There were no notable hematological or biochemical findings. The tumor markers carcinoembryonic antigen (CEA; 2.6 ng/mL) and cancer antigen 125 (15.9 U/mL) levels were within the normal ranges; however, squamous cell carcinoma (SCC) level was slightly elevated at 3.2 ng/mL.

**FIGURE 2:**
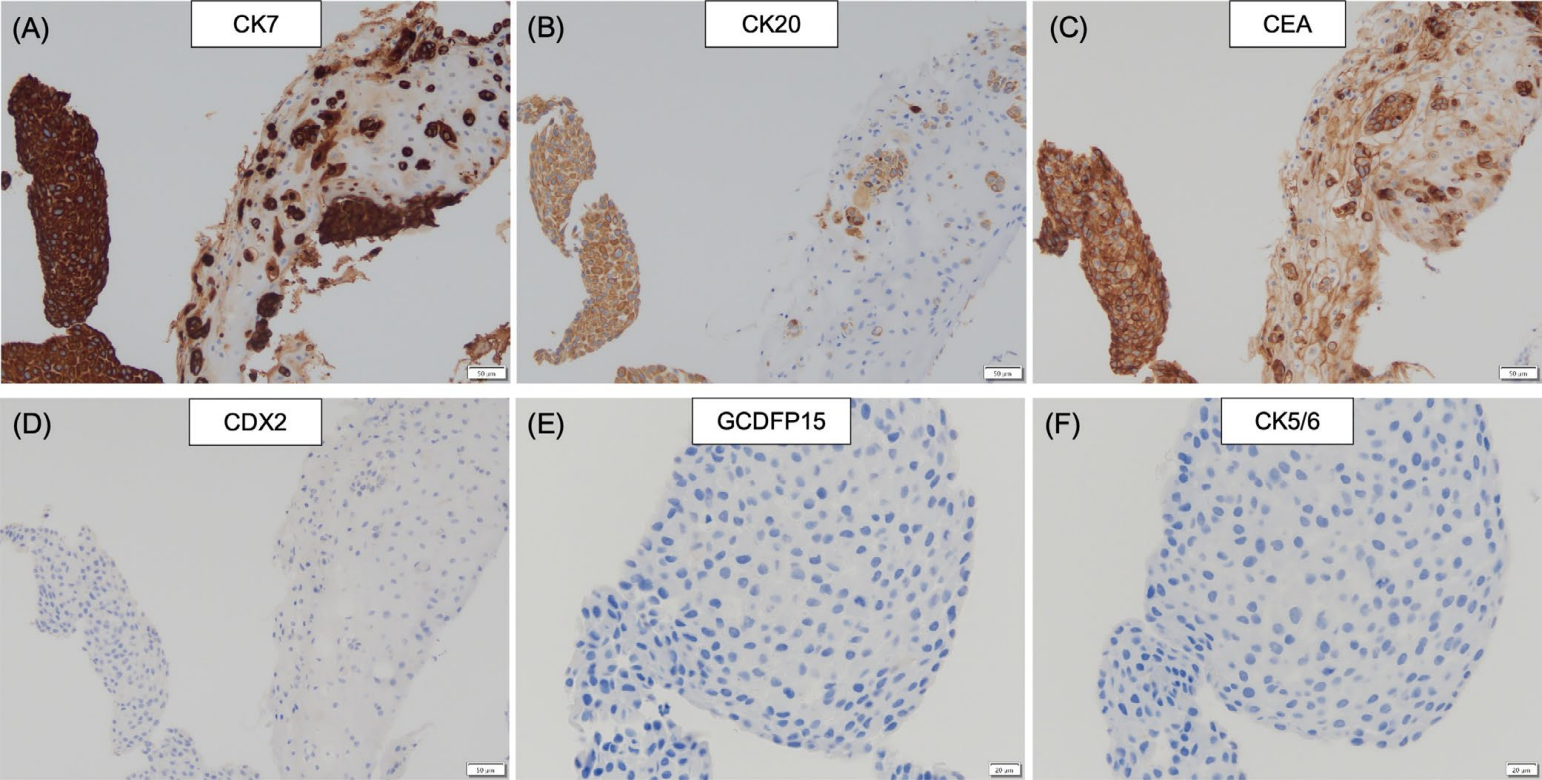
Immunohistochemistry of biopsy specimens (A) Cells are positive for CK7. CK7 immunohistochemical stain; 20× magnification. (B) Positive for CD20. CK20 immunohistochemical stain; 20× magnification. (C) Positive for CEA. CEA immunohistochemical stain; 20× magnification. (D) Negative for CDX2. CDX2 immunohistochemical stain; 20× magnification. (E) Negative for GCDFP15. GCDFP15 immunohistochemical stain; 10× magnification. (F) Negative for CK5/6. CK5/6 immunohistochemical stain; 10× magnification.

Rectal endoscopy revealed no obvious masses in the rectum or anus. Abdominal computed tomography (CT) image showed a 5‐cm mass with contrast in the right vulvar area (Figure 1E). Swelling of the lymph nodes was observed in both inguinal regions, with the right lymph nodes being considerably larger, suggesting lymph node metastasis of the tumor. Multiple enlarged abdominal para‐aortic lymph nodes were observed, suggesting intraperitoneal lymph node metastasis (Figure 1F). There were no other obvious tumors in the intestines, bladder, uterus, or vagina. Cytological examination of natural urine was not performed, whereas cytological examination of catheterized urine did not reveal any atypical cells. Histological examination included biopsies from the center and edge of the right vulvar tumor. The tissue biopsied from the center of the tumor contained atypical Paget cells with sheet‐like clusters of nucleoli (Figure 1B). The tissue biopsied from the edge showed the progression of atypical cells in a small alveolar or scattered pattern within the non‐neoplastic squamous epithelium (Figure 1C,D). Immunohistochemical staining of the tumor tissue was positive for cytokeratin (CK)20, CK7, CEA, and cytokeratin 5.2 (CAM5.2) (Figure 2A–D), and negative for p40, caudal‐type homeobox transcription factor 2 (CDX2), gross cystic disease fluid protein 15 (GCDFP15), and CK5/6 (Figure 2E–H). A summary of the immunohistochemical results is presented in Table 1. Owing to her advanced age and dementia, treatment was difficult, and she was left untreated. Seven months after the initial diagnosis, she developed Trousseau syndrome owing to symptoms of cerebral infarction and died of the original disease. A CT scan performed immediately before the patient's death revealed lung and intraperitoneal lymph node metastases of the tumor, but no obvious masses in the urinary or digestive tract. The examination also showed no masses in the urethra or anus other than a vulvar mass.

**TABLE 1:** Summary of immunohistochemistry.

Primary site		Primary EMPD	Secondary EMPD				
IHC	Positive cells	Vulva	Anal	Rectal	Bladder	Gynecology	Vulva (this case)
CK7	Squamous/luminal epithelium, mesothelium	+	+	−	+	−/+	+
CK20	Intestinal/transitional epithelium, Merkel	−	+	+	+	−	+
CEA	Adenocarcinoma	+	+	+	−/+	+	+
CDX2	Mucous epithelium	+	+	+	+	−/+	−
GCDFP15	Apocrine epithelium	+	−	−	−	−	−
CK5/6	Spindle/squamous cell	−	−	−	+	−/+	−

Abbreviations: CDX2, caudal‐type homeobox transcription factor 2; CEA, carcinoembryonic antigen; CK, cytokeratin; EMPD, extramammary Paget's disease; GCDFP15, gross cystic disease fluid protein 15; IHC, immunohistochemistry.

The diagnosis was vulvar adenocarcinoma associated with EMPD, and the tumor, of stage IVB determined according to the International Federation of Gynecology and Obstetrics (FIGO) 2021 [6], was determined to have metastasized to the abdominal cavity and bilateral inguinal lymph nodes. No obvious primary lesions were identified outside the vulva, and immunohistochemical staining of the tumor ruled out SCC, digestive tract disease, or urothelial disease. Immunohistochemical staining revealed that the tumor was not a primary EMPD, but rather a secondary EMPD, indicating a vulvar adenocarcinoma with pagetoid spread.

## 3 Discussion

In this case, the diagnosis was vulvar adenocarcinoma associated with EMPD. However, clinical findings and immunohistochemical staining revealed that this was a very rare case of secondary EMPD presenting with pagetoid spread with no obvious primary lesion other than that in the vulva. If immunohistochemical staining reveals pagetoid spread in the vulva, the condition is classified as secondary EMPD necessitating an investigation to identify the primary site outside the vulva. However, in this case, no lesions were identified outside the vulva, suggesting an extremely rare scenario where the pagetoid spread may have originated from the vulva itself.

More than 80% of vulvar cancers are SCCs, followed by melanomas. The rare histological types include basal cell carcinoma, verrucous carcinoma, adenocarcinoma associated with EMPD, Bartholin's adenocarcinoma, and sarcoma. Our patient has adenocarcinoma associated with EMPD (FIGO 2021) [6].

EMPD occurs in areas rich in apocrine glands in the genitals and perianal skin, and can be divided into primary and secondary EMPD. Primary EMPD is thought to originate from the apocrine sweat glands, as it occurs most frequently in the vulva, axillae, and perianal areas. Secondary EMPD is characterized by the progression of anal canal and rectal cancers to the anal skin or by the progression of bladder and gynecological cancers to the vulvar skin, showing signs of intraepidermal carcinoma, a phenomenon called pagetoid spread. Therefore, secondary EMPD is not strictly associated with Paget's disease, but rather with the progression of other cancers to the skin of the vulva. The proportion of secondary EMPD in the vulva is approximately 15%–20%. The most common primary lesions are anal, rectal, and bladder cancers, in that order [7]. Primary EMPD and secondary EMPD, which presents with pagetoid spread, have similar clinical and pathological findings, but the treatment approaches are completely different; therefore, their differentiation is important [2]. The most important prognostic factor is the depth of invasion. Tumors deeper than 1 mm have a poor prognosis, and spreading to lymph nodes or other tissues beyond the skin increases the risk [8]. Compared to primary EMPD, secondary EMPD has a poor prognosis because the primary lesion is often highly malignant and progressive [9]. Therefore, it is necessary to perform biopsy and radiology promptly to establish a definitive diagnosis [10]. Primary and secondary EMPD present with similar skin lesions that are difficult to differentiate using hematoxylin–eosin staining alone. Therefore, immunohistochemical staining must be performed to make a diagnosis.

Carcinoembryonic antigen (CEA) test and periodic acid‐Schiff (PAS) stain are commonly used for the immunohistochemical examination of EMPD, and GCDFP15 and CK20 are used to differentiate primary from secondary EMPD. GCDFP15 expression is positive in sweat, mammary, and salivary glands, but negative in the digestive tract [11]. CK20 is expressed in the digestive tract epithelium, bladder epithelium, Merkel cells, digestive tract adenocarcinoma, bladder transitional cell carcinoma, and Merkel cell carcinoma [12]. Immunohistochemical staining has shown that primary EMPD is GCDFP15 positive [13] and CK20 negative [7, 14]. In contrast, secondary EMPD that progresses from rectal cancer, anal cancer, or bladder transitional cell carcinoma is GCDFP15 negative and CK20 positive [7, 15, 16]. GCDFP‐15 is reported to be positive in up to 90% of primary EMPD cases, whereas CK20 is reported to be positive in up to 95% of secondary EMPD cases [17]. In this case, the tumor was GCDFP15 negative and CK20 positive, with no obvious abnormalities in the digestive or urinary tracts despite the diagnosis of secondary EMPD.

There has been a report of cases in which, similar to the present case, CK20 expression was positive and GCDFP15 expression was negative and no masses were found in the rectum or anal canal, leading to a diagnosis of primary perianal Paget's disease or pagetoid spread of anal and rectal canal cancer [3]. In that report, the tumors were positive for CDX‐2 expression in five of six cases. *CDX2* is a homeobox gene that encodes an intestine‐specific transcription factor that is expressed in the nuclei of intestinal epithelial cells from the duodenum to the rectum. CDX2 expression is positive in primary and metastatic colorectal cancer and is considered a more specific and sensitive marker than CK20 [18]. The frequency of CDX2 positivity has been reported to be 2% in primary EMPD and 33% in secondary EMPD [19, 20], with higher rates in secondary EMPD due to the prevalence of lesions in the gastrointestinal tract. In the present case, CDX2 expression was negative, and there were no abnormalities in the tissue examination of the lower gastrointestinal tract; therefore, the possibility of lower gastrointestinal disease was considered low.

The immunohistochemical test parameters used to differentiate between primary and secondary EMPD, other than GCDFP15 and CK20, are described below. A combination of CK7 and CK20 has been used; primary EMPD tends to be CK7 positive and CK20 negative, whereas secondary EMPD tends to be both CK7 and CK20 positive [9]. In the present case, CK7 and CK20 expression was positive, which is consistent with the characteristic of secondary EMPD. In addition, the immune profile of urothelial carcinoma has been reported to be CK7+/CK20+/GCDFP15− [1], which is consistent with the finding in the present case. However, in this case, the expression of CK5/6 was negative, although it is known that it is typically remarkably positive in urothelial carcinoma. Based on the macroscopic findings and catheter urine cytology, the possibility of a primary urinary tract tumor was judged to be low [21].

Vulvar EMPD is extremely rare, accounting for only 1%–2% of genital tumors [22, 23]. EMPD is often diagnosed when the disease is already quite advanced, due to its subtle and unclear clinical findings [24]. The disease generally progresses slowly; however, in some cases, it may spread deeply to lymph nodes and, rarely, hematogenously [25]. In this case, the patient presented with advanced disease, with metastasis to the pelvic lymph nodes. In most EMPD cases, there is a discordance between the gross and histologic margins of the disease. This necessitates the use of biopsy techniques for accurate evaluation before surgery. A preoperative vulvovaginal examination tool called “clock mapping” was previously proposed to predict the invasiveness and spread of EMPD [4]. Clock mapping consists of multiple vulvovaginal biopsies performed in different areas, both in the superficial vulvoperineal region and in the central deep vaginal region. Recently, a modified clock mapping biopsy technique has been proposed that allows for a more accurate evaluation [26]. When localized lesions are present, accurate preoperative evaluation is essential, as the removal of the lesion is considered the gold standard treatment. However, in this case, considering the patient's advanced age and dementia, treatment was not indicated. Therefore, examination of the lesion margin was not performed.

Secondary EMPD is a condition in which cancer in an organ adjacent to the skin migrates intraepithelially to reach the epidermis and presents as intraepidermal cancer. It is reasonable to consider that there is a continuity between adjacent visceral cancer and Paget's disease. In this case, the lesion was only in the vulva, and there was no anal, rectal, or bladder cancer. Paget disease is divided into three categories: Paget disease secondary to anal or rectal adenocarcinoma, Paget disease secondary to urothelial neoplasia, and Paget disease secondary to adenocarcinomas or related tumors of other sites [27]. In this case, the cause was determined to be vulvar cancer, which falls under the “Paget disease secondary to adenocarcinoma of other sites” category. Although this has not been reported previously, we determined that the vulvar adenocarcinoma had progressed to the surrounding normal squamous epithelium and presented as pagetoid spread, as determined using histopathological examination. We considered the possibility that vulvar adenocarcinoma was the primary site and that secondary EMPD had progressed to the surrounding vulvar squamous epithelium.

EMPD is extremely rare; hence, there are limited data on recommended treatment options. Currently, surgical resection is indicated for invasive vulvar Paget's disease; however, due to the blurred contours, the margins are often positive, and recurrences are frequent [28]. In cases of poor general condition or inability to undergo surgical treatment, conservative management can be considered as an alternative. Among these treatments, CO_2_ laser, photodynamic therapy, imiquimod cream, radiotherapy, or chemotherapy may help improve the quality of life [25, 29]. In this case, vulvar cancer metastasized to the lymph nodes in both inguinal regions, abdominal para‐aortic lymph nodes, and lungs, and the patient died of the primary disease 7 months after the initial diagnosis, with a poor prognosis.

The unique feature of this case is that it represents a rare instance of vulvar adenocarcinoma with pagetoid spread, a finding consistent with secondary EMPD on immunohistochemical staining. However, no primary site was identified outside the vulva. Due to the patient's advanced age, dementia, and the advanced stage of vulvar cancer, aggressive treatment was not pursued. Additionally, no biopsy or surgical treatment was performed to evaluate the lesion preoperatively, which limited the ability to make a definitive diagnosis.

## 4 Conclusion

This is a rare case of vulvar adenocarcinoma with pagetoid spread, a finding of secondary EMPD on immunohistochemical staining, but no other primary site was found besides the vulva. In secondary EMPD with pagetoid spread, identifying other primary sites and conducting an accurate preoperative evaluation are crucial. However, as demonstrated in this case, the primary site can sometimes be the vulva itself, with the disease progressing locally.

## Author Contributions

**Kazuaki Nishimura:** conceptualization, investigation, methodology, writing – original draft, formal analysis, writing – review and editing. **Seiji Kagami:** visualization, resources, review and editing. **Haruka Kajio:** investigation, visualization, resources. **Tamaki Wada:** visualization, resources. **Naoyuki Toki:** visualization, resources. **Kiyoshi Yoshino:** conceptualization, resources, review and editing, supervision.

## Ethics Statement

This is not a medical study involving human participants. A written explanation was provided to ensure that the patient's family would not be disadvantaged.

## Consent

The patient's family provided written informed consent for the publication of this case report and accompanying images.

## Conflicts of Interest

The authors declare no conflicts of interest.

## Data Availability

The data that support the findings of this study are available on request from the corresponding author. The data are not publicly available due to privacy or ethical restrictions.
